# Photosynthetic Induction Characteristics in Saplings of Four Sun-Demanding Trees and Shrubs

**DOI:** 10.3390/plants14010144

**Published:** 2025-01-06

**Authors:** Qiuping Liu, Wei Jin, Liying Huang, Danfeng Wang, Kedong Xu, Yunmin Wei

**Affiliations:** 1 Institute of Translational Medicine, College of Life Science and Agronomy, Zhoukou Normal University, Zhoukou 466001, China; liuqp06@163.com (Q.L.); jinw877@126.com (W.J.); 13285936238@139.com (L.H.); 2 Key Laboratory of Plant Genetics and Molecular Breeding, Zhoukou Normal University, Zhoukou 466001, China; wdf19911025@live.com

**Keywords:** photosynthetic induction, photosynthetic limitations, woody species, initial stomatal conductance, *Ficus macrocarpa* L.

## Abstract

Light serves as the unique driving force of photosynthesis in plants, yet its intensity varies over time and space, leading to corresponding changes in the photosynthetic rate. Here, the photosynthetic induction response under constant and fluctuating light was examined in naturally occurring saplings of four sun-demanding woody species, *Eucalyptus*. *Ficus macrocarpa* L., *Hibiscus syriacus* L. and *Ficus carica* L. We aimed to find out the relations among gas exchange parameter adaptions among different species during photosynthetic induction. The net photosynthetic rates (*A*) versus time course curves were sigmoidal or hyperbolic after the dark-adapted leaves were irradiated by continuous saturated light. Compared with other species, *Ficus carica* L. have the largest net photosynthesis rate, stomatal conductance to CO_2_ (*g*_sc_), and the maximum carboxylation rate (*V*_cmax_) at both the initial and steady photosynthetic state. The initial *g*_sc_ (*g*_sci_) was as much as sixfold higher compared to the other shrub, *Hibiscus syriacus* L. The time required to reach 90% of *A* (t_A90_) was 7–30 min; t_A90_ of *Ficus carica* L. and *Ficus macrocarpa* L. were lower than that of the other two species. The time required to reach 90% of *g*_sc_ (t_gsc90_) significantly lagged behind t_A90_ among species. Biochemical induction was fast in leaves of *Ficus carica* L., as about 4 min were needed to reach 90% of *V*_cmax_, while the other species needed 7–18 min. Correlation analysis showed that the t*_gsc_*_90_ was the main factor in limiting t_A90_, especially for *Eucalyptus* spp. and *Hibiscus syriacus* L.; *g*_sci_ was negatively correlated with t_gsc90_ among species. Moreover, time-integrated limitation analysis revealed that *g*_sc_ still accounted for the largest limitation in constraining *A* of *Eucalyptus* spp. and *Hibiscus syriacus* L. and *Ficus macrocarpa* L. Overall, the findings suggest that to enhance the carbon gain by woody species under naturally dynamic light environments, attention should be focused on improving the rate of stomatal opening or initial stomatal conductance.

## 1. Introduction

In natural and agricultural ecosystems, plants are frequently exposed to dynamically changing light environments due to intermittent cloud movement and cover, wind-induced leaf movements, variations in solar angle, or other atmospheric factors [1,2]. Fluctuating light irradiation in plant leaves induces dampened photosynthetic oscillations, resulting in photosynthesis rarely achieving a stable state. However, the photosynthetic response to light fluctuations (such as a sudden shift from low light to high light irradiation) is not instantaneous. According to the classic photosynthetic biochemical model by Farquhar et al. [3], photosynthetic rate (*A*) was limited by the three rates: (1) the maximum rate of Rubisco catalyzed carboxylation (Rubisco-limited); (2) the regeneration of ribulose-1,5-bisphosphate (RuBP), controlled by electron transport rate (RuBP-limited), and (3) the regeneration of RuBP, controlled by the rate of triose-phosphate utilization (TPU-limited). As a result, it typically exhibits an induction period characterized by a gradual increase of *A* overtime to a stable state rate [4]. Furthermore, plants require time to adjust to the transition from low light or darkness to high light irradiations. Recent studies showed that, compared to the ideal situation where *A* could reach the stable state immediately after the light fluctuation, the damped *A* oscillation in fluctuating light affects carbon gain in plants [5,6]. Additionally, the photosynthetic organs could be damaged due to the imbalance of light–dark reactions in the photosynthetic process [7,8]. Therefore, further investigation into the physiological responses of plant photosynthesis under dynamic light conditions is necessary.

When leaves previously in darkness are illuminated, the *A* cannot reach the highest level instantly (since several processes underlying photosynthesis are activated or deactivated slowly) but needs to increase to a steady-state rate gradually over a period of time. This process is defined as photosynthetic induction [9]. Depending on plant species, light conditions, and environmental conditions, the duration of photosynthetic induction can range from a few minutes to tens of minutes [10]. Currently, the underlying mechanisms of photosynthetic induction have been extensively studied. According to the current understanding, photosynthetic induction is affected by three main physiological processes, i.e., the induction rate of photosynthetic electron transport in the thylakoid membrane, activation of Calvin–Benson cycle enzymes, and stomatal opening [11]. Taken together, photosynthesis is limited by diffusional and biochemical constraints [12]. Diffusional constraints are primarily attributed to stomatal opening, while biochemical constraints arise due to the light and carbon reactions driving *A* [13]. The proportion of these two factors is closely related to plant species and is also affected by plant genotype and external environmental conditions. Based on analyses of photosynthetic induction limitations in rice, the primary limitation during induction was biochemical limitation rather than stomatal limitation [14]. Since Rubisco enzymes are susceptible to being deactivated under shade or dark conditions, the reactivation of Rubisco enzymes under high light conditions might be crucial for inducing *A* during transitions from darkness to light [15]. Studies on different genotypes of wheat showed that the reduction of the instantaneous response of the maximum carboxylation rate (*V*_cmax_) during photosynthetic induction resulted in a 15% reduction in the net carbon gain [6]. In other crops (such as cassava), as well as tropical trees or shrubs, photosynthetic induction is strongly constrained by stomatal conductance to CO_2_ (*g*_sc_) [16,17,18]. Similarly, in a range of horticultural crops, changes induced by photosynthesis are also primarily driven by stomatal traits, e.g., by initial *g*_sc_ and the rate of stomatal opening during the induction [19]. In addition, the interactions between stomatal opening and biochemical processes may have a combined effect on photosynthetic induction. For instance, slow opening of the stomata can delay induction by starving Rubisco of CO_2_ in dark and low-light irradiation conditions [20].

Woody species occupy a specific ecological niche in plant community [21], as they are exposed to rapid and alternating changes in solar radiation over their vegetation period, except for some uppermost leaves of the canopy, which may experience prolonged periods of sufficient light under steady-state conditions [22]. In such cases, a significant portion of CO_2_ assimilation occurs under fluctuating light conditions. Early studies have highlighted the significance of various photosynthetic limiting factors on wood photosynthesis under stable light conditions [23,24,25]. Under fluctuating light conditions, species exhibit variation in the traits that dominate photosynthetic induction; however, there is limited information on inter-specific differences in induction properties of woody species in China. In the present study, we aimed to elucidate the acclimation of dynamic *A* in important common trees (*Eucalyptus* spp. And *Ficus macrocarpa* L.) and shrubs (*Hibiscus syriacus* L. and *Ficus carica* L.) in the coastal region of China. Our hypothesis proposed that these species obtained a typical response to photosynthetic induction. The traits relevant to the photosynthetic induction process were measured, and limitation analyses were conducted to determine the main limiting factors of each species to enhance the photosynthetic performance of woody species efficiently under naturally dynamic light environments.

## 2. Results

### 2.1. Photosynthetic Induction Responses

**Figure 1:**
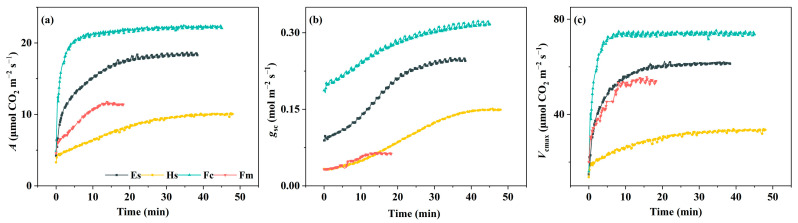
Dynamic photosynthetic traits of *Eucalyptus* spp. (Es), *Hibiscus syriacus* L. (Hs), *Ficus macrocarpa* L. (Fc), and *Ficus macrocarpa* L. (Fm) during photosynthetic induction at 1000 μmol m^−2^ s^−1^ photosynthetic photon flux density (PPFD). (**a**) the net photosynthetic rate (*A*) response curves; (**b**) the stomatal conductance to CO_2_ (*g*_sc_) response curves; (**c**) the maximum carboxylation rate (*V*_cmax_) response curves.

After illuminating, *A* of four woody species increased rapidly in the initial stage of induction, followed by a more gradual increase until a steady state was reached (Figure 1). Similarly, induction of *g*_sc_ and *V*_cmax_ showed typical responses, except that the *g*_sc_ curve was observed a certain delay in the initial stage. During the induction, *A*, *g*_sc_, and *V*_cmax_ varied significantly between accessions; the accession *Ficus macrocarpa* L. had the highest photosynthetic performance all the time, while *Hibiscus syriacus* L. had the lowest. Interestingly, *Ficus carica* L. could reach a steady state faster than the other accessions in less than 20 min (Figure 1).

### 2.2. Dynamic and Steady Changes of Photosynthetic Traits During Photosynthetic Induction

**Figure 2:**
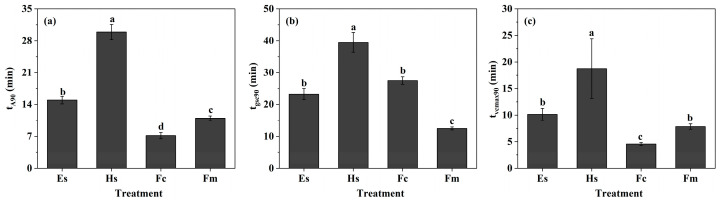
Dynamic photosynthetic parameters of different plants during photosynthetic induction. t_A90_, t_gsc90_, and t_vcmax90_ were the time (min) to reach 90% of photosynthetic induction, full stomatal opening, and biochemical activation. (**a**), value of t_A90_ among the species; (**b**), value of t_gsc90_ among the species; (**c**), value of t_vcmax90_ among the species. Different letters indicate significant differences among the species. Data are means ± SE (n = 3–6). *Eucalyptus* spp. (Es), *Hibiscus syriacus* L. (Hs), *Ficus macrocarpa* L. (Fc), and *Ficus macrocarpa* L. (Fm).

Rates of photosynthetic induction increased during the episode of light induction (Figure 1), affecting the time to reach 90% of full induction. Further analysis of the time required for induction states of species showed that *Hibiscus syriacus* L. had the longest time (29.9 min) to achieve 90% of photosynthetic induction (t_A90_), which was significantly higher than that of *Eucalyptus* spp. (15.0 min), *Ficus macrocarpa* L. (11.0 min), and *Ficus carica* L. (7.2 min) (Figure 2). A similar trend was observed in time to reach 90% of biochemical activation (t_Vcmax90_), whereas the time was generally lower than that of t_A90_ (Figure 2). Compared with t_A90_, the t_Vcmax90_ of *Hibiscus syriacus* L., *Eucalyptus* spp., *Ficus macrocarpa* L. and *Ficus carica* L. decreased by 37.3%, 32.1%, 28.6% and 36.3%. By contrast, stomata opened slower during the induction, and the time to reach 90% of stomatal opening (t_gsc90_) was higher than that of t_A90_ and t_Vcmax90_. In particular, the t_gsc90_ of *Hibiscus syriacus* L. reached 39.5 min, which was almost 3.2-fold longer than that of *Ficus macrocarpa* L (Figure 2).

Moreover, a multiple regression model was employed to compare the effect of t_gsc90_ and t_Vcmax90_ on t_A90_ (Table 1). For *Eucalyptus* spp. and *Hibiscus syriacus* L., the regression coefficient of t_gsc90_ was much higher than that of t_Vcmax90_, suggesting a stronger influence in determining t_A90_; On the contrary, t_Vcmax90_ exerted a greater impact on t_A90_ in species *Ficus macrocarpa* L., and especially on *Ficus carica* Linn., of which the regression coefficient of t_vcmax_90 was almost 10-fold higher than t_gsc90_ (Table 1).

**Figure 3:**
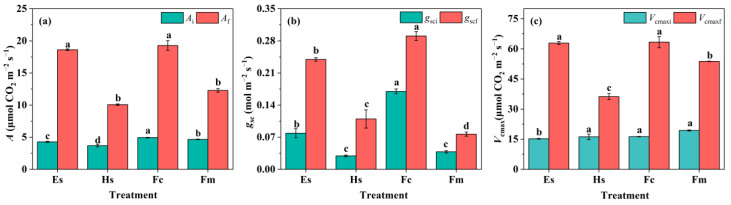
Steady-state photosynthetic parameters of different plants during photosynthetic induction. (**a**), *A*_i_ and *A*_f_ were the steady-state *A* of the last 1 min of low light induction period (100 μmol m^−2^ s^−1^ PPFD) and high light induction period (1000 μmol m^−2^ s^−1^ PPFD), respectively. (**b**), *g*_sci_ and *g*_scf_ were the steady-state *g*_sc_ of the last 1 min of low light induction period (100 μmol m^−2^ s^−1^ PPFD) and high light induction period (1000 μmol m^−2^ s^−1^ PPFD), respectively. (**c**), *V*_cmaxi_ and *V*_cmaxf_ were the steady-state *V*_cmax_ of the last 1 min of low light induction period (100 μmol m^−2^ s^−1^ PPFD) and high light induction period (1000 μmol m^−2^ s^−1^ PPFD), respectively. Different letters indicate significant differences among the species. Data are means ± SE (n = 3–6). *Eucalyptus* spp. (Es), *Hibiscus syriacus* L. (Hs), *Ficus macrocarpa* L. (Fc), and *Ficus macrocarpa* L. (Fm).

Steady-state gas exchange parameters at the end of photosynthetic induction were further analyzed. We found that the highest t_A90_ of *Hibiscus syriacus* L. was accompanied by the lowest steady-state *A* at both 100 (*A*_i_) and 1000 PPFD (*A*_f_) inducing periods (Figure 3). On the contrary, *Ficus carica* L. and *Eucalyptus* spp. achieved the highest *A*_f_ with shorter induction time, which was 19.3 and 18.6 μmol m^−2^ s^−1^, respectively. Moreover, the steady-state stomatal conductance (*g*_scf_) and biochemical activity (*V*_cmaxf_) of *Ficus carica* L. and *Eucalyptus* spp. were higher than that of *Hibiscus syriacus* L. and *Ficus macrocarpa* L. in response to the light fluctuating course (Figure 3).

### 2.3. Stomatal and Biochemical Limitations During Photosynthetic Induction

**Figure 4:**
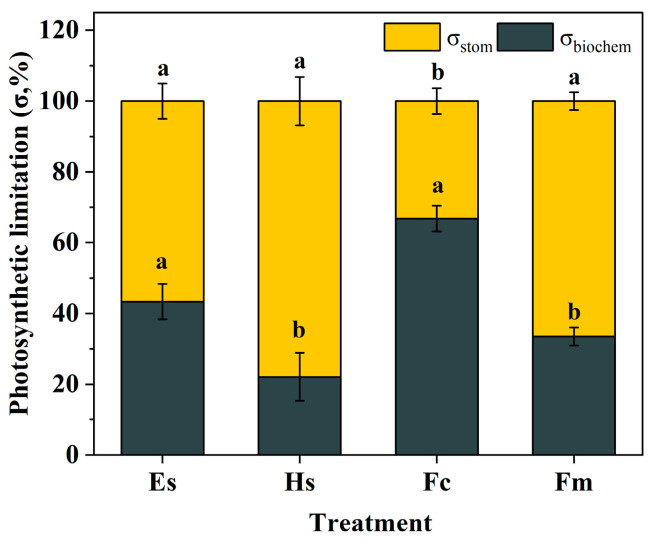
The impact on photosynthetic induction due to the stomatal limitation (σ_stom_) and biochemical limitations (σ_biochem_). Data are means ± SE (n = 3–6). Different letters indicate significant differences among the species. *Eucalyptus* spp. (Es), *Hibiscus syriacus* L. (Hs), *Ficus macrocarpa* L. (Fc), and *Ficus macrocarpa* L. (Fm).

The limitation model quantified the degree of stomatal opening and biochemical process that constrained *A* during the episode of photosynthetic induction. This is a complex parameter that the sum of these limitations is 100%, as they are calculated in relation to the total limitation for *A*, referenced by gaseous diffusion and biochemical state. Analyses of the limiting factors for the induction of photosynthesis have shown that a high proportion of stomatal conductance limitation (σ_stom_) was the key factor for *A* of *Hibiscus syriacus* L. in the induction process (Figure 4). In particular, the σ_stom_ accounting for approx. 78% of the total limitation, significantly higher than that of *Ficus macrocarpa* L. (66%) and *Eucalyptus* spp. (57%) (Figure 4). On the contrary, biochemical capacity limitation (σ_biochem_) has the pivotal constrain on *A* of *Ficus carica* L. for more than 60%, which was significantly increased by twofold and onefold compared to *Hibiscus syriacus* L. and *Ficus macrocarpa* L (Figure 4).

### 2.4. Relationship Between Parameters in Relation to Photosynthetic Induction

**Figure 5:**
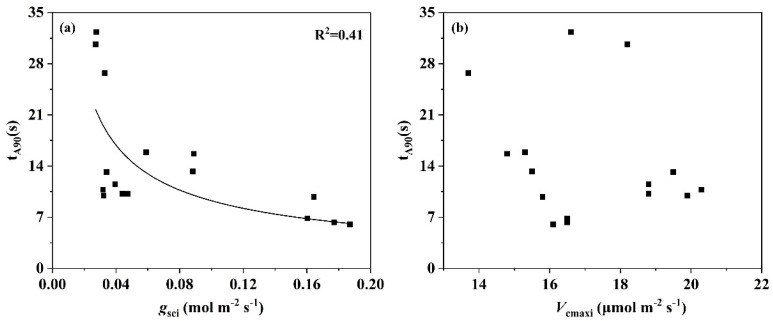
t_A90_ in relation to *g*_sci_ (**a**) and *V*_cmaxi_ (**b**). Filled squares display values (n = 3–6) for the four species considered in the present study. Data were fitted in by a regression analysis in considering all species.

Considering the different gas exchange parameters that determine the time to reach 90% steady-state photosynthesis, correlations in varying degrees between t_A90_ and *A*_i_ (R^2^ = 0.85), *A*_f_ (R^2^ = 0.36), and *g*_sci_ (R^2^ = 0.41) were observed in the present study, while no statistical correlation was found between t_A90_ and *V*_cmaxi_ (Figure 5). Specifically, the value of t_A90_ dropped rapidly with the elevation of *A*_i_ and *A*_f_ compared with the slow response of t_A90_ to the varying of *g*_sci_.

## 3. Discussion

Eco-physiological studies require knowing the photosynthetic characteristics of different species across different environmental settings. Among these, enhancing dynamic photosynthesis generally has the maximum carbon gains for plants, especially for sun-demanding plants [13,20,26,27]. In the present study, four sun-demanding woody species, including two trees and two shrubs commonly found in coastal areas of China, were selected, and the photosynthetic induction characteristics were compared using the same experimental setup. Findings suggested that increasing the initial stomatal conductance would be more beneficial for dynamic photosynthesis than the rate of stomatal opening or biochemical capacities for these species.

Typically, time courses of photosynthetic induction curves are either sigmoidal or hyperbolic [26]. Here, the induction curves were similar among species, rising rapidly within 5–10 min, followed by a rise to the steady-state value, except for *Hibiscus syriacus* L., which showed a gradual increase throughout the induction (Figure 1a). As Allen & Pearcy, the shape of the photosynthetic induction curve was mainly determined by *g*_sci_ since the dark-adapted treatment before induction basically eliminated the variation of Rubisco enzyme traits [16]. Along with this, we found that the *g*_sci_ of *Hibiscus syriacus* L. significantly reduced in comparison to *Ficus carica* L., presenting a sigmoidal induction type (Figure 1b). The result was in line with Kirschbaum & Pearcy, who suggested that a sigmoidal induction type is observed when *g*_sci_ is initially low, causing a relatively slow increase in *A* during induction [28]. Similarly, *Ficus macrocarpa* L., which have a lower *g*_sci_, showed a weak sigmoidal type of induction (Figure 3). However, the rate of biochemical induction seemed to be faster in *Ficus macrocarpa* L. leaves, which significantly contributed to a further increase in *A* compared to *Hibiscus syriacus* L (Figure 3). The average time for trees, such as Eucalyptus spp. and Ficus macrocarpa L., to reach 90% of *A* was short, around 10–15 min. In contrast, the difference of t_A90_ between the two shrubs was more than fourfold, in which t_A90_ in *Hibiscus syriacus* L. was approx. 30 min (Figure 2), indicating a plasticity in the rate of photosynthetic induction of shrubs. In the ecological community, the induction time was affected by many factors and showed an irregular change. Generally, plants grown in low light induced faster than those grown in high light, manifested by a rapid induction [21,22,29,30]. However, slow induction can also be found across species, lasting up to 1 h or longer, independent of the light requirements of plant growth [31,32]. Zipperlen & Press [33] compared two climax trees grown in different forest light conditions and suggested that species showed a higher induction rate when exposed to medium light or high light, contrary to expectations for shade-tolerance species.

To date, numerous studies have reported on the mechanisms influencing photosynthetic induction, identifying Calvin-cycle enzyme activity, the regeneration of Calvin-cycle intermediates, and stomatal opening rates as the key factors [30,34,35]. Moreover, Kromdijk et al. [36] found a faster relaxation of NPQ was beneficial to CO_2_ assimilation during light transition from high to low intensity. In our study, we found that there was a general increase of t_gsc90_ compared to t_A90_ (Figure 2), suggesting that the stomatal opening rate lags the photosynthetic rate during the induction. Particularly, t_gsc90_ of *Ficus carica* L. increased by approx. 20 min than the t_A90_ (Figure 2). Suwannarut et al. [37] examined *g*_sc_ in ten tropical species and suggested that the stomata of *Ficus carica* L. opened and closed slower when compared with other species, and their opening became progressively slower under a series of light flecks. It can also be drawn from the stomatal induction curve in Figure 1b. Recent studies have often linked stomatal opening rates to stomatal size, noting that smaller l stomates tend to respond more rapidly to an increase in irradiance than the larger stomates [38,39,40], although stomatal opening was co-determined by stomatal density. Further investigation into the factors for the greatly increased t_gsc_ of *Ficus carica* L is warranted. Multiple regression analysis revealed that the inter-specific differences in induction times are likely due to differences in the dynamic responses of these slow-inducing components (Table 1). In comparison with *Ficus carica* L. and *Ficus macrocarpa* L., t_gsc90_ of *Eucalyptus* spp. and *Hibiscus syriacus* L. had a greater influence on the t_A90_. Interestingly, even though the t_gsc_ of *Ficus carica* L. was higher than that of *Eucalyptus* spp., t_gsc90_ was not the key factor limiting the t_A90_, which may be ascribed to its higher *g*_sci_. Studies on varieties of species have confirmed that there is a certain negative correlation between the *g*_csi_ and t_A90_ [39,41,42]. Consistent with these findings, our study demonstrated that as the increase of *g*_sci_, t_A90_ showed a slow decline, while the effect of *V*_cmaxi_ on t_A90_ was slight (Figure 5).

Overall, photosynthetic increased rapidly upon exposure to light (Figure 1 and Figure 2). Within species, photosynthetic induction of *Ficus carica* L. was mainly constrained by biochemical limitation, whereas stomatal limitation accounting for a large proportion of photosynthesis limitation of *Hibiscus syriacus* L. and *Ficus macrocarpa* L, stomatal and biochemical limitations contributed equally to photosynthetic induction of *Eucalyptus* spp. (Figure 4). It is important to note that leaf age could impact these results. Research by Urban et al. [43] compared the limitations during the induction phase in young and mature leaves of poplar and revealed that the rubisco activation limitation was significantly higher in mature leaves than in young leaves. Future research should consider evaluating s photosynthetic induction traits in plant canopies with different leaf ages.

## 4. Materials and Methods

### 4.1. Plant Material and Growth Condition

Two one-year-old common shrubs (*Hibiscus syriacus* L. and *Ficus macrocarpa* L.) and two one-year-old trees (*Eucalyptus* spp. and *Ficus macrocarpa* L.) were grown individually in the outdoor places. Plants were planted in 5 L pots filled with a standard soil matrix, which contains slow-release nitrogen, potassium and phosphorus fertilizer. Before the experiment started, plants were moved to a greenhouse for one week for acclimation. The air temperature was set to 25 °C for 16 h photoperiod and 18 °C for 8 h at night, the relative humidity was 65%, and the light was maintained at above 800 μmol m^−2^ s^−1^ photosynthetic photon flux density (PPFD).

### 4.2. Gas Exchange Measurement

For the measurement, the youngest fully expanded leaf of each plant was placed in a 2 × 6 cm cuvette of Li-6400XT gas-exchange system (Li-Cor, Lincoln, NE, USA); plants were selected and measured randomly from 9:00–11:30. Within the cuvette, the flow rate of air was 500 μmol s^−1^, air temperature was 25 °C, PPFD was 1000 μmol m^−2^ s^−1^, CO_2_ concentration was maintained at 400 μmol mol^−1^ and the relative humidity was controlled to 60%. Once the steady state was achieved, net photosynthetic rate (*A*), CO_2_ concentration in the intercellular spaces (*C*_i_), and leaf stomatal conductance (*g*_s_) were recorded. Since *g*_s_ is the stomatal conductance to water vapor, it is expressed in terms of stomatal conductance to CO_2_ (*g*_sc_) in subsequent calculations, which is calculated as *g*_sc_ = *g*_s_/1.6.

### 4.3. Photosynthetic Induction Measurement

To assess the response of gas exchange to a dynamic irradiance, leaves were first allowed to adapt in the low light of 100 μmol m^−2^ s^−1^ PPFD for steady and followed by 1000 μmol m^−2^ s^−1^ PPFD until photosynthetic parameters achieved a steady state. In these conditions, gas exchange measurements were logged every second for the first 1 min and every 5 s thereafter. The steady-state *A* and *g*_sc_ of the last 1 min of the low light induction period were expressed as *A*_i_ and *g*_sci_, while *A*_f_ and *g*_scf_ were equal to the final *A* and *g*_sc_ at the last 1 min of high light induction. For each plant, 3–6 biological replicates were used, and all calculations were performed on single replicates.

Photosynthetic induction was calculated according to the following:

(1) $$\mathrm{IS}=\frac{A-A_{i}}{A_{f}-A_{i}}$$

The induction of *g*_sc_ over the same duration was also calculated by replacing *A*, *A*_i_, and *A*_f_ with *g*_sc_, *g*_sci_, *g*_scf_, respectively. t_A90_ and t_gsc90_ were defined as the time to reach 90% of the difference between initial and maximum values of photosynthetic induction and stomatal opening time, respectively.

According to Farquhar et al. [3], at low light or the initial stage of high light induction, *A* was limited mainly by RuBP regeneration. Since the activation of RuBP regeneration occurs rapidly, Rubisco activation began to limit *A* (or co-limit with RuBP regeneration) in quite a short time, along with the duration of irradiance. Therefore, it is assumed that *A* is primarily limited by Rubisco throughout the induction, and the mesophyll CO_2_ conductance was infinite [6,44,45]. The maximum carboxylation rate of Rubisco (*V*_cmax_) could be calculated as follows:

(2) $$V_{\mathrm{cmax}}=\frac{\left(A+R_{d}){(C}_{i}+K_{m}\right)}{\left(C_{i}-\Gamma^{*}\right)}$$

where *R*_d_ is the rate of respiration in the light, Γ* is the CO_2_ compensation point in the absence of mitochondrial respiration, and *K*_m_ is the effective CO_2_ Michaelis–Menten constant for Rubisco.

Using the above equation, *V*_cmax_ and biochemical activation time could be calculated at each time point. Accordingly, steady-state *V*_cmax_ at the last 1 min of induction under low light (*V*_cmaxi_) and high light (*V*_cmaxf_), as well as the time to reach 90% of the difference between initial and maximum values of *V*_cmax_ (t_Vcmax90_), were obtained.

### 4.4. Limitation Analysis

According to Sakoda et al. [20], altering the induction kinetics of mesophyll conductance would have little impact on *A* following a dynamic light. Hence, only the relative limitation of stomatal and biochemical capacity on *A* during photosynthetic induction was considered here [46], and the *A* variation between final state and steady-state *A* (d*A*) can be modeled by:

d*A* = d*A*_stom_ + d*A*_biochem_ (3)

where d*A*_stom_ and d*A*b_iochem_ are the stomatal and biochemical components that limited *A*, which can be calculated as:

(4) $${dA}_{\mathrm{stom}}=\frac{\partial A}{{\partial g}_{\mathrm{sc}}}dg_{\mathrm{sc}}$$

(5) $${dA}_{\mathrm{biochem}}=\frac{\partial A}{{\partial V}_{\mathrm{cmax}}}dV_{\mathrm{cmax}}$$

where d*g*_sc_ and d*V*_cmax_ are the variations between final state and steady-state *g*_sc_ and *V*_cmax_, respectively.

As *A* = *g*_sc_(*C*_a_ − *C*_i_), combined with Equation (2), gives the partial derivatives as follows:

(6) $$\frac{\partial A}{{\partial g}_{\mathrm{sc}}}=\frac{\frac{A}{g_{\mathrm{sc}}^{2}}\left((V_{\mathrm{cmax}}-R_{d}\right)-A)}{{(V}_{\mathrm{cmax}}-R_{d})(\frac{1}{g_{\mathrm{sc}}})+(C_{a}+K_{m})-2(\frac{1}{g_{\mathrm{sc}}})A}$$

(7) $$\frac{\partial A}{{\partial V}_{\mathrm{cmax}}}=\frac{\left(C_{a}-\Gamma^{*}\right)-A(\frac{1}{g_{\mathrm{sc}}})}{{(V}_{\mathrm{cmax}}-R_{d})(\frac{1}{g_{\mathrm{sc}}})+(C_{a}+K_{m})-2(\frac{1}{g_{\mathrm{sc}}})A}$$

The relative limitation of stomatal (σ_stom_, dimensionless) weighted by *A* could be calculated as:

(8) $$\sigma_{\mathrm{stom}}=\frac{\int dA_{\mathrm{stom}}dt}{\int dA_{\mathrm{stom}}dt+\int dA_{\mathrm{biochem}}dt}$$

σ_stom_ was the estimate of the proportion of stomatal limitation on A during the time scale of photosynthetic induction. While the proportion of biochemical limitation (σ_biochem_, dimensionless) was quantified as: σ_biochem_ = 1 − σ_stom_.

### 4.5. Statistical Analysis

The data are mean ± standard error (SE). Significant differences in parameters were tested using SPSS 25.1 by one-way ANOVA. Significant relationships between parameters were tested by linear regressions. Graphical depiction and regression analyses were conducted with Origin Pro 2020.

## 5. Conclusions

In the present study, we found significant differences in the induction status and time of gas exchange parameters among the discussed sun-demanding species, especially between the two shrubs. Regarding the time course of photosynthesis induction, the induction time of *Ficus carica* L. was shorter, of which the photosynthetic parameters were also maintained at a higher level. This rapid response to high light and high maintenance state of *Ficus carica* L. are important for photosynthetic carbon fixation under fluctuating light environments. For other species, such as *Hibiscus syriacus* L., the increased induction times occurred because of stomatal behavior; the stomatal characteristics, especially *g*_sci_, are the key factors in limiting photosynthetic induction. Further efforts should be paid to the responses of individual stomata to fluctuating light inductions of woody species, such as the diurnal or seasonal changes, which may contribute to a positive plant carbon balance and total carbon gain.

## Figures and Tables

**Table 1:** Multiple regression analysis of photosynthesis (t_A90_) as a function of t_gsc90_ and t_vcmax90_ (t_A90_ = a + b1 × t_gsc90_ + b2 × t_vcmax90_) based on the data of different species.

Species	Intercept	Regression Coefficient		
	a	b1	b2	R^2^
*Eucalyptus* spp.	−0.81	0.74	0.32	0.96
*Hibiscus syriacus* L.	−31.74	1.27	0.63	0.57
*Ficus carica Linn.*	−9.38	0.22	2.29	0.78
*Ficus macrocarpa* L.	6.85	0.10	0.57	0.67

## Data Availability

Data are contained within the article.
